# The added value of online user-generated content in traditional methods for influenza surveillance

**DOI:** 10.1038/s41598-018-32029-6

**Published:** 2018-09-18

**Authors:** Moritz Wagner, Vasileios Lampos, Ingemar J. Cox, Richard Pebody

**Affiliations:** 1 Public Health England, London, UK; 2 University College London, London, United Kingdom; 3 London School of Hygiene and Tropical Medicine, London, United Kingdom; 4 Department of Computer Science, University College London, London, UK; 5 Department of Computer Science, University of Copenhagen, Copenhagen, Denmark

## Abstract

There has been considerable work in evaluating the efficacy of using online data for health surveillance. Often comparisons with baseline data involve various squared error and correlation metrics. While useful, these overlook a variety of other factors important to public health bodies considering the adoption of such methods. In this paper, a proposed surveillance system that incorporates models based on recent research efforts is evaluated in terms of its added value for influenza surveillance at Public Health England. The system comprises of two supervised learning approaches trained on influenza-like illness (ILI) rates provided by the Royal College of General Practitioners (RCGP) and produces ILI estimates using Twitter posts or Google search queries. RCGP ILI rates for different age groups and laboratory confirmed cases by influenza type are used to evaluate the models with a particular focus on predicting the onset, overall intensity, peak activity and duration of the 2015/16 influenza season. We show that the Twitter-based models perform poorly and hypothesise that this is mostly due to the sparsity of the data available and a limited training period. Conversely, the Google-based model provides accurate estimates with timeliness of approximately one week and has the potential to complement current surveillance systems.

## Introduction

Most of the influenza surveillance schemes currently used by Public Health England (PHE) and other national and international public health organisations are based on data from established health systems and thus are skewed towards only a certain subset of overall influenza cases within the population, i.e. those that result in the use of healthcare systems. To improve timeliness and geographical granularity of existing systems work has been undertaken in recent years to establish syndromic surveillance systems that provide data on a daily basis from a range of health systems. Using online content as a source for such systems offers rapid access to understanding the health status of a wider range of the population with the potential of including the bottom part of the disease population pyramid, which represents those who may not seek medical attention^1^.

Use of web-based data to support influenza surveillance has been gaining increasing interest in recent years^2–4^. A multitude of research efforts have established the surveillance potential of data sources from social media^5–10^, search queries^11–15^, or health websites^16^. Nevertheless, there has also been considerable critique of such methods, most famously that involving Google Flu Trends^12^, which was unable to consistently estimate the level of influenza activity when compared to traditional data sources^17–19^. However, such concerns have been alleviated by identifying and addressing the deficiencies of these early approaches^13^. Thus, when considering the incorporation of online data sources within existing public health surveillance systems, their added value needs to be assessed carefully^3^.

The majority of studies that produce time series estimates for influenza activity based on web data, make use of mean out-of-sample estimation error or correlation-type metrics to assess their value when compared to traditional data^5,6,8–14,16^. From a public health perspective, accurate time series estimates are crucial, but there are other important indicators that determine appropriate public health responses throughout an influenza season. The timing of the onset and peak of the influenza season, for example, can inform when to best initiate vaccination or antiviral campaigns. When considering the added value of novel surveillance systems, they must be analysed with respect to their prospective usage by public health bodies.

Machine learning approaches that use online user-generated content–specifically, Twitter or Google search data–were recently developed to estimate influenza-like illness (ILI) rates in England^13,15,20^. The models produce daily and weekly estimates of ILI rates for England and at NHS regional levels. Models based on Twitter and Google data were employed and trained on Royal College of General Practitioner (RCGP) ILI rates. These are based on GP consultations from a sentinel network of approximately 100 practices in England, which covers a registered population of about 1 million people^21^. They represent the weekly incidence rate of ILI cases (consultations) per 100,000 patients registered with eligible practices during that week.

The aim of this paper is to evaluate these two new surveillance systems, which are based on online content, by comparing them to established influenza surveillance approaches and assessing their added value at PHE. The evaluation adopts an adapted CDC approach to evaluating surveillance systems^22–24^.

## Methods and Materials

Two approaches were implemented that utilise online content based on Twitter posts and Google search query data. The following sections outline the models used and the data involved.

### ILI models based on Twitter posts

Twitter data consist of daily random samples which make up approximately 1% of all tweets originating from England. To produce ILI estimates, frequency rates of a set of influenza related *n*-grams (phrases containing *n* words) are extracted and input to the model, which consists of a nonlinear Gaussian Process (GP) model trained on historical RCGP data. As RCGP data are given on a weekly basis, daily model estimates are based on Twitter data of the last 7 days. The statistical framework for this originates from and is described in detail in a previous study^20^. The supervised model is trained on historical RCGP ILI rates (March 2012-August 2015) separately on a national and NHS regional level using Twitter data originating from the relevant areas. Thus, five supervised Twitter models are respectively available for: England, North England, South England, Midlands & East England and London. Daily or weekly (based on Twitter data of the past 7 days) ILI estimates are produced for all these models from the start of the 2015/16 influenza season onwards.

### Modelling ILI rates based on Google search queries

The available Google data consist of daily estimates of the proportion of searches for specific queries, based on randomised samples of approximately 15% of all search queries performed in England. The latter have been obtained via a private Google Health Trends API. Applying a similar approach as for Twitter, frequency rates of relevant search queries are input to a GP model to produce ILI estimates^13,15^. The model is trained on historical RCGP ILI rates (January 2007-August 2015) on a national level, as search queries were available at a national level only. Daily or weekly (based on Google data of the past 7 days) ILI estimates are produced from the start of the 2015/16 influenza season onwards.

## Performance evaluation metrics

The proposed system is evaluated with respect to its statistical validity as a measure for ILI rates and in terms of the quality of the data involved. Statistical validity is assessed by comparing estimates produced by each model with ILI indicators from traditional surveillance sources. This involves the use of existing surveillance sources, specifically RCGP ILI rates and Data Mart laboratory confirmed cases and a variety of statistical measures.

The Twitter and Google models are built to specifically estimate RCGP ILI rates and are thus compared to official RCGP ILI rates (number of GP ILI consultations per 100,000 GP registered population) on a national and subnational level for the 2015/16 influenza season. Data from week 40 in 2015 up to and including week 20 in 2016 are used, which corresponds to the period from the week ending 04/10/2015 to the week ending 15/05/2016. Model performance is measured with a focus on predicting the onset, overall intensity, peaks and duration of the influenza season.

To further explore the underlying dynamics involved, RCGP ILI rates by age and laboratory confirmed cases by influenza type for the 2015/16 influenza season are also compared to model estimates. Note that neither Google nor Twitter data are available by age-group and model estimates did not target a particular influenza type.

### Overall intensity

The following measures are used to quantify the overall fit of the supervised models’ estimates to RCGP data throughout the 2015/16 influenza season:

- Pearson correlation (r)
- Mean Square Error (MSE)
- Root Mean Square Error (RMSE)
- Mean Absolute Error (MAE)
- Mean Absolute Percentage Error (MAPE)
- Mean Error (ME)
- Max Error and Week of Max Error: Largest absolute error compared to RCGP data and the corresponding week
- Max Percentage Error and Week of Max Percentage Error: Largest absolute percentage error and the corresponding week. The absolute percentage error here refers to the absolute error divided by the weekly RCGP ILI rate.

### Onset

The following measures are used to quantify the ability of the national supervised models’ to accurately estimate the starting week of the 2015/16 influenza season as given by the RCGP data:

- Alert week: The first week of the influenza season with a rate above the pre-epidemic threshold. Thresholds were calculated using the Moving Epidemic Method (MEM) based on both national and subnational RCGP data of the previous 6 influenza seasons^25^.
- Time difference: The difference between the alert week of the RCGP data and the alert week resulting from the model estimates. A negative value means the model predicted a later alert week, while a positive value indicates an early alert week.

### Peaks

The following measures are used to quantify the ability of the supervised models to accurately estimate the peaks that occurred in RCGP data during the 2015/16 influenza season both in terms of timeliness and intensity. In order to determine the 2nd peak, only estimates beyond +/−2 weeks of the 1st peak were considered.

- Magnitude of 1st peak-to-peak difference: Difference in ILI rate between the 1st peak in the RCGP data and the 1st peak in the model.
- Temporal offset of 1st peaks: Difference in weeks between the week of the 1st peak in the RCGP data and the week of the 1st peak in the model estimates.
- Magnitude of 1st peak-to-model difference (same week as RCGP estimate): Difference in ILI rate between the 1st peak in the RCGP data and the model estimate of the same week.
- Magnitude of 2nd peak-to-peak difference: Difference in ILI rate between the 2nd peak in the RCGP data and the 2nd peak in the model estimates.
- Temporal offset of 2nd peaks: Difference in weeks between the week of the 2nd peak in the RCGP data and the week of the 2nd peak in the model estimates.
- Magnitude of 2nd peak-to-model difference (same week as RCGP estimate): Difference in ILI rate between the 2nd peak in the RCGP data and the model estimate of the same week.

### Age and influenza type

To investigate possible biases towards certain age groups or influenza types, the model ILI estimates are also compared to RCGP ILI rates by age and laboratory confirmed cases by influenza type (A and B) for the 2015/16 influenza season. The laboratory confirmed cases are based on the Respiratory Data Mart system, which incorporates test results from all PHE laboratories and a number of NHS labs that took part in the extended PHE pandemic Influenza testing network in 2009/10^26^. The data consist of the percentage of laboratory confirmed cases by influenza type. As the supervised models do not produce estimates by age or influenza type, they are not directly comparable to these data sets. Nevertheless, similarities or differences can offer some insights into what sort of factors might be influencing estimates produced by the supervised models. To assess this, the Pearson correlations between the model estimates and RCGP ILI rates by age and Data Mart estimates by influenza type (A and B) during the 2015/16 influenza season were examined. Here data from week 40 in 2015 up to and including week 16 in 2016 are used, which corresponds to the period from the week ending 04/10/2015 to the week ending 17/04/2016. This is done on a national level only due to data availability.

## Statistical assessment of web-based ILI models

The following section presents a comparison between the various model estimates for ILI and other surveillance sources (i.e. RCGP, Data Mart) graphically and using the performance metrics outlined previously. This is done separately for the Twitter models nationally and subnationally and the Google model.

### Twitter models

#### National level

**Figure 1:**
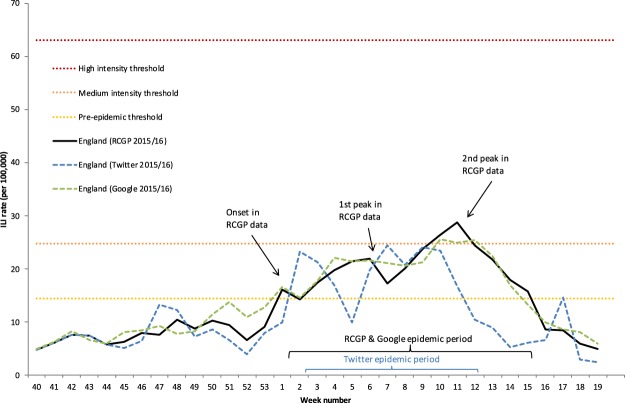
RCGP ILI estimates with overlaid national Twitter (blue) and Google (green) supervised model ILI estimates by week number during the 2015/16 influenza season. Thresholds were calculated using the Moving Epidemic Method based on national RCGP ILI estimates of the previous 6 influenza seasons^25^.

**Figure 2:**
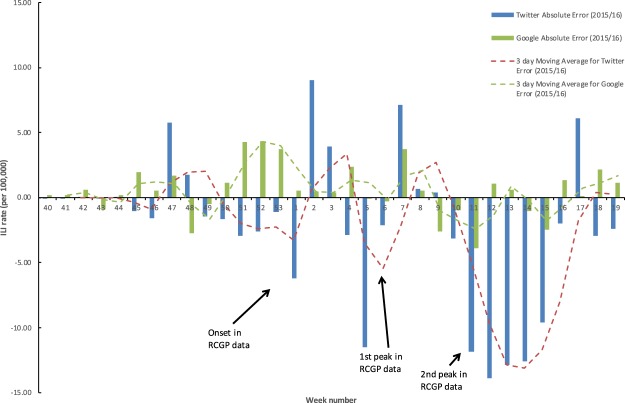
Absolute errors between RCGP data and the national Twitter (blue) and Google (green) supervised model ILI estimates by week number during the 2015/16 influenza season including their 3 day moving averages.

On a national level, the Twitter supervised model exhibits a moderately good fit for the overall intensity with a Pearson correlation of 0.67 compared to the RCGP ILI rates (Table 1). The onset is estimated to be week 2, a week later than the RCGP data which breached the pre-epidemic threshold in week 1 that marks the start of the influenza season (Table 1 and Fig. 1). Additionally, the Twitter model produces an early peak during week 47, which does not go above the pre-epidemic threshold, but does represent the largest percentage error throughout the season (Table 1). There were two peaks observed in the RCGP data during the 2015/16 season initially in week 6 and then week 11 and for the Respiratory Data Mart data in week 5 for influenza A(H1N1)pdm09 and then week 11-12 for influenza B^27^. The first peak in week 2 in the Twitter model is estimated 4 weeks prior to the first peak in the RCGP data, but demonstrates a similar level of intensity (Table 1). The second peak in week 11, on the other hand, was barely detected by the model (Table 1). Looking at Table 2, there is a clear indication that the Twitter estimates are closer to laboratory confirmed cases for influenza type A than type B. Note that the majority of influenza A consisted of H1N1pdm09 during the 2015/16 season. Furthermore, RCGP ILI rates for younger and middle aged adults (Table 3), particularly those 15–44 and 45–64 years of age, have much stronger correlations (0.67–0.68) with the Twitter estimates compared to ILI rates for young children and the elderly (0.20–0.53). There is a negative mean error in the national Twitter estimates, although looking at the error values over time, it appears most of this is due to the underestimation of the second peak, where the peak-to-model error is −11.87 (41.36%) (see Table 1 and Fig. 2).

**Table 1:** Measures for the performance of the Twitter supervised models (national and subnational) and the Google model (national) in estimating the overall intensity, onset and peaks of the 2015/16 influenza season when compared to RCGP ILI rates (national and subnational).

	Subnational level				National level	
	London Twitter	Midlands and East Twitter	North Twitter	South Twitter	England Twitter	England Google
**Overall intensity**						
r	0.37	0.37	0.40	0.31	0.67	0.96
MSE	69.46	70.06	71.72	131.5	36.57	3.86
RMSE	8.33	8.37	8.47	11.47	6.05	1.96
MAE	6.28	5.95	6.13	7.83	4.27	1.47
MAPE	39.10%	45.55%	40.85%	45.31%	29.95%	14.10%
ME	−2.58	−5.43	−5.13	−6.99	−2.17	0.54
Max Error	18.05	19.95	23.42	32.81	13.88	4.32
Week Max Error	47	13	11	12	12	52
Max Percentage Error	214.88%	84.53%	83.05%	207.50%	75.72%	66.12%
Week of Max Percentage Error	47	13	11	46	47	52
**Onset**						
Alert week	1	47	51	3	2	1
Time difference	2	−2	0	−1	−1	0
**Peaks**						
Magnitude of 1st peak-to-peak difference	−0.55 (2.04%)	−3.57 (17.76%)	3.93 (18.36%)	−2.92 (10.25%)	1.32 (6.03%)	0.15 (0.68%)
Temporal offset of 1st peaks	12	2	4	2	4	2
Magnitude of 1st peak-to-model difference (same week as RCGP estimate)	−4.79 (17.74%)	−15.63 (77.76%)	−13.35 (62.38%)	−18.82 (66.04%)	−2.12 (9.68%)	−0.31 (1.42%)
Magnitude of 2nd peak-to-peak difference	6.05 (21.61%)	−11.86 (48.61%)	−7.81 (27.70%)	−25.24 (66.07%)	−4.28 (14.91%)	−3.16 (11.01%)
Temporal offset of 2nd peaks	6	5	4	2	4	1
Magnitude of 2nd peak-to-model difference (same week as RCGP estimate)	−15.03 (53.68%)	−19.81 (81.19%)	−23.42 (83.05%)	−32.81 (85.89%)	−11.87 (41.36%)	−3.86 (13.45%)

**Table 2:** Pearson correlations between national supervised Twitter and Google models and Data Mart laboratory confirmed cases by influenza type during the 2015/16 influenza season.

	Influenza Type A	Influenza Type B	All Data Mart laboratory confirmed cases
England Twitter	0.83	0.24	0.74
England Google	0.82	0.68	0.95

**Table 3:** Pearson correlations between national supervised Twitter and Google models and RCGP ILI rates by age during the 2015/16 influenza season.

	Age (years)						
	<1	1–4	5–14	15–44	45–64	65–74	75+
England Twitter	0.20	0.42	0.53	0.67	0.68	0.39	0.22
England Google	0.53	0.72	0.79	0.96	0.91	0.70	0.45

#### Subnational level

Comparing the subnational Twitter models to the equivalent subnational RCGP ILI rates, low Pearson correlations are observed ranging from 0.31–0.40. Furthermore, error values remain large across all regions, e.g. the maximum percentage error is ranging from 83.05% to 214.88%, especially when estimating the second peak of the influenza season in week 11 (Table 1). The onset is estimated fairly well across all regions with no more than 2 weeks difference compared to the onsets determined by the RCGP data (Table 1). The two peaks of the 2015/16 influenza seasons are also observable in the RCGP regional data. The regional Twitter models, however, estimate neither of the peaks very well with large errors occurring particularly during the second RCGP peak for all regions, where the percentage errors for ILI estimates in the same week range from 53.68% to 85.89% (Table 1). As with the national estimates, large negative mean errors indicate that the Twitter models tend to underestimate RCGP ILI rates, but again this is mainly due to especially low estimates during the second peak (Table 1).

### Google model

The overall fit of the Google model to RCGP data is very good with a high Pearson correlation of 0.96 and only small error values (Table 1). The largest error (absolute error of 4.32 and percentage error of 66.12%) was found during week 52 of the 2015/16 influenza season, which corresponds to the week ending on 27/12/2015. The onset of the influenza season (based on MEM) was estimated accurately without error (Table 1). The Google model estimated the two peaks of activity well, but they occurred slightly earlier compared to the ones observed through the RCGP data (2 and 1 weeks early for the first and second peak, respectively). Additionally, the second, larger peak was slightly underestimated, but not significantly with a percentage error of 13.57% in the model’s ILI estimate during the week of the second peak in the RCGP data (Table 1). There exists a stronger correlation between Google estimates and type A laboratory confirmed cases (0.82) than type B laboratory confirmed cases (0.68) (Table 2). In addition, correlations with RCGP ILI rates are particularly high for the age groups 15–44 (0.96) and 45–64 (0.91), whereas for children and the elderly the correlations are smaller (age groups <1, 1–4 and 5–14 have correlations of 0.53, 0.72 and 0.79, respectively, while for age groups 65–74 and 75+ the correlations are 0.70 and 0.45, respectively) (Table 3). There is a small positive mean error in the Google estimates (Table 1). Looking at the error values over time, this may be due to some overestimates around week 52 (Fig. 2).

## Assessment of Data Quality

Several sources of bias need to be taken under consideration when evaluating the model estimates. Biases may be present in the web-based data (Google search, Twitter), the statistical modelling frameworks, but also in the health surveillance data used for evaluation (i.e. RCGP, Data Mart).

### Causal links, word sense disambiguation and web data sparsity

The deployed Twitter and Google models are purely statistical and as such they do not aim to capture the causal path that triggers a particular search query. For example, when a user searches for “fever”, we cannot always be certain that this particular user has this symptom; it may be a different reason that caused this search query. We assume that, on average, the probability of having a fever when searching for it is consistent enough to be captured by a statistical model with a degree of noise. Our current models, however, can, to a certain extent, disambiguate between the use of words in different semantic contexts, e.g. they can disambiguate between the semantic meaning of “fever” and “Saturday Night fever” or “Bieber fever”^15^, using a framework based on word embeddings^28^. In addition, with access limited to only a proportion of geo-located Tweets (1%), online data can be sparse, especially on a regional/sub-national level. The amount of Google search queries available (15%) appears to represent an adequately dense data set, but currently is not available at a regional level. In this particular study, there was also a discrepancy in the length of the training periods used for the models (March 2012-August 2015 for the Twitter models, January 2007-August 2015 for the Google model). For the Twitters models, the shorter training period is more likely to entail biases of certain influenza seasons.

### RCGP and Data Mart data

Both supervised models are currently trained on RCGP data and thus are biased towards this data source with the major drawback being the fact that RCGP ILI rates are based solely on the people with ILI symptoms who visited their GP. This excludes the majority of people with ILI in the general population who commonly do not seek medical assistance. In addition, any biases present in RCGP data are likely to be carried over to model estimates. An analysis of the RCGP sentinel network, for example, showed an over-representation of the cohort in the 25–44 year age group, whilst people of white ethnicity and less deprived people were under-represented, when compared to national data^21^. Hence, for influenza types that disproportionally affect the elderly and/or children, the supervised models might underestimate ILI rates. On the other hand, overestimates might be produced for influenza types that tend to affect younger adults.

The percentage of laboratory confirmed influenza cases given by the Data Mart system is useful in giving insights into the different influenza subtypes involved throughout an influenza season and how their dynamics change. Nevertheless, like the RCGP data, it is limited to patients that seek medical attention. Thus, it is not necessarily representative of the overall incidence of influenza in the general population, especially if certain influenza subtypes result in more or less severe disease.

### Demographics

Users of both Twitter and Google data are mostly adults. This can, however, also include posts and search queries done by adults about their children. In addition, the data are biased towards users that have access to the Internet and use Google or Twitter, although the Internet penetration in England is very high with approximately 90% of adults in the UK reporting Internet usage^29,30^.

The majority of Twitter users are aged 15–44 with a higher proportion likely to be situated in urban areas. In 2015 the UK was estimated to include 13.1 million Twitter users^31^. Google users, on the other hand, are likely to be less biased towards younger adults and with not only the volume of search queries performed daily, but also the sample rate (15%) of the data available for the model being significantly higher than that of Twitter posts (1%), it is likely to provide a significant reduction in noise compared to the Twitter data available. Neither of the data sources include explicit demographic information of the users, although these may be inferred to highlight biases of certain estimates^32–35^.

### Pathogenic

The models used were trained on specific influenza seasons (March 2012-August 2015 for the Twitter models, January 2007-August 2015 for the Google model) and thus are biased towards the pathogens of those seasons, which vary in their transmission dynamics. For H1N1pdm09 for example, there is some immunity in over 65 year-olds and models trained on H1N1pdm09 seasons might include such a demographic bias.

## Discussion

Two models were evaluated with respect to RCGP and Data Mart data. The models were assessed on a national and subnational level for the 2015/16 influenza season with the overall aim of evaluating the added value of Twitter and Google data in combination with machine learning techniques to traditional influenza surveillance systems. This was done by analysing each model’s ability to estimate ILI rates through a number of statistical indicators, whilst considering biases involved in the data sources used.

The Twitter supervised model performs relatively poorly at a national level with a Pearson correlation of 0.67 and even less so at a subnational level with Pearson correlations ranging from 0.31 to 0.40, when compared to RCGP ILI rates for the 2015/16 influenza season. While the timing of the onset of the influenza season is estimated well by the model, estimates of the overall intensity and the peaks observed in RCGP data were poorer. The underestimation observed during the second peak could be due to the fact that the second peak was predominantly caused by influenza B, which had a stronger effect on children (5–14 years of age) as opposed to Influenza A (H1N1) that had dominated most of the early season and was mainly affecting working adults (15–64 years of age)^27^. The high correlations with influenza type A laboratory confirmed cases and with RCGP ILI rates of younger and middle aged adults observed for the national estimates support this hypothesis. The 25–44 year age band is slightly over-represented in the RCGP sentinel network and this bias will be carried over into any model estimates^21^. At the same time, Twitter users are more prominent in the adult age groups^31^, so the model may have not picked up on the increasing prevalence during the second peak with more activity in children. With only 1% of all Tweets and only a relatively short time series available for training (three flu seasons), the sparsity of the Twitter data at a subnational level is likely to be the main cause for its poor performance. In fact, when using the national estimates of the Twitter model and comparing these to the subnational RCGP estimates, the fit is significantly better showing that influenza transmission patterns are similar on a national and subnational level, something that is to be expected. Further work is needed to understand the performance of the Twitter models, but the volume of Twitter data available, which is limited to geo-located tweets, and the biases involved in Twitter data are discouraging factors. A much larger volume of Twitter data together with more historical data may produce better ILI estimates as various past research efforts have hinted^6,36^.

The Google supervised model provided very accurate estimates when compared to RCGP ILI rates with a Pearson correlation of 0.96. The largest discrepancy in the estimates, which was observed during week 52, includes the Christmas holidays. During this week GPs were only open for 4 days and therefore RCGP ILI estimates are expected to be less accurate and generally lower. Interestingly, despite having a small drop in its ILI rate estimate as well, the Google model gives a higher estimate for this week indicating that it might be picking up cases missed by the RCGP data. The slight underestimation of the second peak in RCGP ILI rates could again be due to the fact that it was predominantly caused by influenza B^27^. The correlations observed with laboratory confirmed cases and RCGP ILI rates by age and the fact that Google users are more prominent in the adult age groups support this hypothesis. Hence, the few discrepancies in the estimates could be explained through biases in the RCGP data either due to closed GP practices or pathogenic changes throughout the season creating a demographic shift. This indicates that the Google supervised model estimates may serve as viable instantaneous ILI estimates throughout the influenza season. Furthermore, the Google model provided some advantages in terms of timeliness compared to RCGP data. As RCGP ILI rates are published through PHE with a delay of 4 days, the supervised model ILI estimates, if accurate, have the potential to offer an early-warning alternative. In combination with current surveillance sources, extensions of such models could provide public health bodies with a broader picture of the true prevalence of influenza during each influenza season and thus inform on the most effective use of resources. Moreover, they offer the opportunity to gain further insights into the transmission dynamics occurring during a particular influenza season, if discrepancies between the Google and RCGP estimates are observed. Due to lack of available data, it was not possible to deploy the model at a sub-national level, something that can possibly provide further added value.

Future work could focus on investigating the geographical spread of disease over time. Currently, for the Twitter models presented, there exists only a breakdown on NHS regional level. Exactly geo-located tweets and Google search queries could become available at a more granular level. This could allow public health agencies to detect localised outbreaks more quickly and offer insights into how influenza spreads. A recent study, using multi-task learning to model regional rates in the US, provides further support for this^37^. Furthermore, the use of geo-located tweets and search queries provides the opportunity to assess the impact of localised interventions, as done for the 2013/14 and 2014/15 LAIV childhood influenza pilots^13,38^. Incorporating automatically inferred user demographics such as age, gender or social class may also be a potential future direction^32–35^. Given the differential effect of specific strains on certain age groups, an accurate characterisation of age-specific burden would be particularly useful, especially when dealing with emerging strains^39^. In general, there is potential to use such data sources not only for surveillance purposes, but also to gain a deeper understanding of disease dynamics. A previous study for example used Twitter data to estimate secondary attack rate and serial interval of ILI^40^.

Work is still required to refine the statistical methods used to deal with issues such as noise, model and data biases and the fact that estimates from web content are not directly based on actual ILI cases. Future work could aim at moving towards unsupervised models that do not depend on traditional surveillance sources, RCGP data in this case, for training purposes. These could infer their own ILI rates based solely on online data originating from the bottom part of the disease population pyramid and thus offer a more independent and complementary data source to traditional surveillance systems. Generally, if implemented for public health purposes, such systems should be considered as on-going, developing tools^3^. Each influenza season offers a new set of data points to test model performance, which may in turn inform subsequent design and development of new statistical approaches. Additionally, it is important to be aware that the nature of the input data may change over time (e.g. changes in user behaviour and evolution of search engines/social media platforms).

Despite issues with biases, discrepancies in model estimates and the fact that estimates from online content are not directly based on actual ILI cases, surveillance based on online user-generated content also offers significant advantages to traditional surveillance sources. Most importantly, systems based on online data provide almost instantaneous estimates of ILI, are cost-effective in terms of implementation and maintenance and offer potential insights into aspects of transmission that may not be captured by traditional surveillance. As this evaluation only involved a single influenza season, it should be continued for further seasons to assess how the models deal with different transmission intensities and circulating strains. With further assessment and development, particularly around the Google model, online user-generated content has the potential of adding value to and complementing existing surveillance systems and thus aiding public health response strategies throughout influenza seasons.

## Data Availability

The data that support the findings of this study were collected by general practices who are Royal College of General Practitioners (RCGP) Research and Surveillance Centre (RSC) network members. These data are available from RCGP RSC. RCGP weekly data are available online (www.rcgp.org.uk/rsc), in weekly and annual reports. Restrictions apply to the availability of individual level data. The data used for the current study were a customised extract and not publicly available, but could be reproduced. The data request form and process for RCGP RSC data is available online (www.rcgp.org.uk/rsc). Data from the Data Mart system are published weekly and annually (https://www.gov.uk/government/collections/weekly-national-flu-reports, https://www.gov.uk/government/statistics/annual-flu-reports) and are available from the authors upon reasonable request and with permission of Public Health England.
